# Dilated cardiomyopathy in patients with hypoparathyroidism: A narrative review

**DOI:** 10.1002/hsr2.1796

**Published:** 2024-01-04

**Authors:** Maryam Abdolmaleki, Laya Ohadi, Saba Maleki

**Affiliations:** ^1^ Shahid Beheshti University of Medical Sciences Tehran Iran; ^2^ School of Medicine Guilan University of Medical Sciences Rasht Iran

**Keywords:** calcium level, calcium supplementation, dilated cardiomyopathy, hypoparathyroidism

## Abstract

**Background:**

Hypoparathyroidism is a rare endocrine disorder characterized by low blood calcium levels, elevated phosphorus levels, and insufficient parathyroid hormone production. It can lead to dilated cardiomyopathy (DCM), a cardiac condition characterized by enlarged ventricles and reduced heart function. This review aims to explore the relationship between hypoparathyroidism and DCM, the impact of calcium on cardiac function, and the potential for DCM reversal with calcium supplementation.

**Methods:**

A comprehensive literature search was conducted using PubMed, Google Scholar, and relevant keywords and Mesh terms. Case reports evaluating dilated cardiomyopathy in patients with Hypoparathyroidism were included in the study. Additionally, references cited in each study were carefully examined to identify relevant reports. The cases included in the review were analyzed, and common cardiac manifestations, diagnostic approaches, and management were identified.

**Results:**

DCM in hypoparathyroidism presents with symptoms of heart failure, reduced ejection fraction, and impaired left ventricular function. Laboratory tests show low serum calcium levels and elevated phosphate levels. Prompt diagnosis and treatment with calcium and vitamin D supplementation can lead to improvements in cardiac function.

**Conclusion:**

Hypoparathyroidism‐induced DCM is reversible with timely calcium and vitamin D supplementation. Patient compliance with prescribed medications and supplements is crucial to prevent and manage cardiac complications. Regular follow‐up check‐ups and monitoring of calcium levels can aid in early detection and improve patient outcomes. Educating patients about the importance of treatment adherence can significantly reduce the risk of developing DCM and other cardiac symptoms associated with hypoparathyroidism. Routine follow‐up of DCM among patients with endocrine disorders is recommended.

## 1 INTRODUCTION

Hypoparathyroidism is an uncommon hormonal deficiency condition characterized by low blood calcium levels, elevated phosphorus levels, and insufficient parathyroid hormone (PTH). It presents with symptoms like tingling sensations, muscle cramps, seizures, bronchospasm, and heart rhythm disturbances.^1^ Acquired hypoparathyroidism, primarily caused by anterior neck surgery, accounts for approximately 75% of cases, while autoimmune disease affecting the parathyroid glands or multiple endocrine glands is the second most common cause. Other rare conditions, such as infiltrative disorders and radiation exposure, can also lead to acquired hypoparathyroidism.^2^ Hypoparathyroidism can lead to complications related to imbalances in calcium‐phosphate levels, resulting in an elevated risk of mortality, renal diseases, infections, and cardiovascular disease (CVD). Lower levels of plasma calcium are associated with an elevated likelihood of developing CVD including dilated cardiomyopathy (DCM).^3^

DCM is a cardiac disorder where the left ventricle or both ventricles enlarge and have reduced contraction, without being caused by excessive pressure, volume overload, or coronary artery disease. Although treatment advancements have improved outcomes, some DCM patients remain vulnerable to sudden cardiac death and refractory heart failure.^4^ Hypoparathyroidism‐induced hypocalcemia, although rare, can cause DCM, which is reversible. Calcium plays a vital role in cardiac function by initiating excitation and contraction of cardiac muscle fibers. Hypocalcemia negatively impacts cardiac contractility, impairs myocardial contraction, and increases the risk of arrhythmias and prolonged QT interval.^5^ Calcium initiates the contraction cycle by binding to troponin C within the cardiac muscle cell. Voltage‐sensitive L‐type calcium channels trigger the release of calcium from the sarcoplasmic reticulum. Hypocalcemia can impair this process, leading to reduced contractility and left ventricular (LV) systolic dysfunction.^6^ Calcium supplementation can reverse DCM due to hypocalcemia, but if the condition becomes severe, permanent structural damage may occur. Timely identification and treatment are vital for minimizing complications and enhancing outcomes.^7^

This review aims to examine the link between hypoparathyroidism and DCM, exploring the effects of hypoparathyroidism on cardiac function, the role of calcium in cardiac contractility, and the potential for reversing DCM with calcium supplementation. Understanding the association between hypoparathyroidism and DCM is crucial for clinicians and healthcare providers. It allows for the early recognition of potential cardiac complications in individuals with hypoparathyroidism. Prompt diagnosis and treatment can improve outcomes, prevent irreversible cardiac damage, and enhance the quality of life for affected patients. It also underscores the potential for reversibility and encourages proactive healthcare practices to improve the overall well‐being of affected individuals and reduce complications.

## 2 METHODS

A comprehensive literature search was conducted using the PubMed and Google Scholar databases to identify relevant articles on DCM in hypoparathyroidism. The search included keywords such as “hypoparathyroidism,” “dilated cardiomyopathy,” “hypocalcemia,” and “parathyroid hormone.” Articles published in English were included, and only one case report was excluded due to the non‐English language. The search was limited to the available publications in PubMed and Google Scholar up to July 2023 which have been demonstrated in Table 1.

**Table 1:** This table demonstrates case reports with hypoparathyroidism associated with dilated cardiomyopathy (DCM).

Study Year Author	Age	Gender	Symptoms	Diagnosis	Treatment
Daniella et al.^8^	38	Female	Shortness of breath, muscle cramp Distended jugular vein, gallop sound, and crackling rales in lungs	Echocardiogram showed cardiomyopathy with reduced ejection fraction	After treating calcium levels after 3 months chambers size was normal
Jung et al.^9^	50	Female	Dyspnea, orthopnea, and rales in auscultation	Prolonged QT interval, cardiomegaly with pulmonary congestion in CXR, and reduced EF in Echo	Normalizing symptoms after treating with calcium
Lam et al.^10^	37	Female	Cramps, facial spasm, heart irregularities, dyspnea, epigastric pain, and edema	Dilation and dysfunction of left ventricles, MR, TR, PHTN in Echo	Intravenous calcium, oral calcium supplements, and vitamin D
Babu and Sameer^11^	70	Female	Paresthesia, cough, exertional breathlessness, bilateral basal capitations with rhonchi in respiratory examination	Echo demonstrated severe DCM with congestive HF, MR, TR, and AR prolonged QT interval in electrocardiogram (ECG)	Intravenous calcium gluconate with oral vitamin D
Vlot et al.^5^	68	Female	Intermittent dyspnea, tachy‐cardia, and tachypnea	Pleural effusion in CXR TEE showed dilated left ventricle, severe dysfunction, MR, and TR	B‐blocker, ACE inhibitor, spironolactone, calcium, and vitamin D were prescribed
Vlot et al.^5^	59	Female	Dyspnea, ankle edema, and systolic murmur	TEE revealed reduced EF, MR, and TR Leftward axis and prolonged QT interval and ST depression in V4‐V6 was found in ECG	Vitamin D, intravenous and oral calcium supplementations
Parepa^12^	40	Female	Syncope, dyspnea, murmur, gallop rhythm, peripheral edema	prolonged QT interval along with negative T waves from V1 to V4 in ECG Echocardiography revealed dilated left chambers and severe global systolic dysfunction of the left ventricle, MR, TR, and PHTN	Intravenous and oral calcium
Benzarouel et al.^13^	29	Female	Dyspnea and generalized edema	TEE dilated LV with global hypokinesia, reduced EF, and MR	Synthyroid medication, calcium and vitamin D
Benzarouel et al.^13^	44	Male	Dyspnea, lower limb edema	TEE revealed dilated cardiomyopathy with a significant impairment in left ventricular systolic function	After correction metabolic disorder, improvement in symptoms were showed
Elikowski et al.^14^	60	Male	Pulmonary edema, pleural effusion	Elevated CPK and BNP Prolonged QT interval in ECG Dilated LV and reduced EF in Echo	Treated with calcium and vitamin D
Valek et al.^15^	26	Female	Paresthesia, upper limb convulsions, acute shortness of breath, malnourished appearance	Prolonged QT interval in ECG Dilated left ventricle and reduced EF Renal and liver dysfunction	Treated with HF therapy, calcitriol, and magnesium supplementation
Valek et al.^15^	74	Male	Dyspnea	Prolonged QT interval in ECG, Echocardiographic examination demonstrated diffuse hypokinesis of nondilated LV with an EF of 44%	Heart failure therapy and calcium supplementations

Abbreviations: AR, aortic regurgitation; CXR, chest X‐Ray; HF, heart failure; MR, mitral regurgitation; PHTN, pulmonary hypertension; TEE, transesophageal echocardiography; TR, tricuspid regurgitation.

The inclusion criteria for the review encompassed studies that investigated the association between hypoparathyroidism and DCM, provided information on the role of calcium and PTH in cardiac function, and reported clinical manifestations and outcomes of DCM in hypoparathyroidism. Peer‐reviewed articles were included, while studies focusing on other forms of cardiomyopathy or lacking relevant data were excluded.

Methods outlined above were employed to review the literature, aiming to provide a comprehensive understanding of the relationship between hypoparathyroidism and DCM.

## 3 DCM PRESENTATION IN HYPOPARATHYROIDISM

Calcium is vital for the heart's proper functioning, controlling its automaticity and excitation‐contraction coordination. When calcium enters myocardial fibers, it triggers the release of stored calcium, leading to increased intracellular levels. This, in turn, enables the interaction between actin and myosin, necessary for muscle contraction. Adequate calcium levels are crucial for optimal heart function.^16^ Hypo‐calcemic cardiomyopathy presents with symptoms resembling heart failure, with most cases showing a reduced ejection fraction (EF), and a smaller proportion experiencing mid‐range or preserved EF. The common manifestations include diffuse LV hypokinesia and sporadic regional wall motion abnormalities. Additionally, patients may exhibit various arrhythmias, such as atrial fibrillation, ventricular tachycardia, and junctional tachycardia.^15^ Here, we have reviewed 11 cases of DCM due to hypoparathyroidism to further investigate the relationship between calcium imbalance and its impact on cardiac function, as well as to examine its clinical presentation and manifestations.

1. A 38‐year‐old woman was admitted to the cardiology department due to severe and persistent shortness of breath (NYHA IV)^1^ for 3 weeks and recurrent muscle cramps over the past 6 months. Her medical history was unremarkable, except for a total thyroidectomy 3 years ago. Upon administration, her vital sign was normal, and she was afebrile. The physical examination revealed left‐sided gallop sounds, distended jugular veins, and crackling rales in her lung bases. Echocardiography showed a dilated and weakened heart muscle (cardiomyopathy) with a reduced EF of 31.4% and increased filling pressure. Laboratory tests showed that the patient had low calcium levels (30 mg/L), decreased free T4 (2.39 pg/mL), and elevated TSH (8.48 μLU/mL). Other tests, including complete blood count, C‐reactive protein, blood glucose, creatinine, and blood ion levels, were within the normal range. After treating her calcium levels with calcium and vitamin D3, in addition to hormone replacement with Levothyroxine, significant improvement in the patient's cardiac symptoms was observed, resulting in the normalization of heart chamber sizes and Left ventricular ejection fraction (LVEF) after 3 months.^8^
2. A 50‐year‐old female with hypoparathyroidism presented dyspnea (NYHA IV) and orthopnea^2^. The dyspnea was improved 5 days before admission. Upon examination, rales were detected, and the electrocardiogram (ECG) revealed a prolonged QT interval. Chest X‐ray showed prominent cardiomegaly with pulmonary congestion, and echocardiography indicated decreased LV function with an EF of 36.5%. Based on her signs and symptoms, she was treated with furosemide injection at first due to heart failure. Lab data demonstrated reduced calcium, magnesium, and ionized calcium levels and increase in phosphorus level. Moreover, intact‐PTH and vitamin D levels were reduced. Parathyroid infiltrating disease and autoimmune disease were role out. Further investigations were conducted to determine the cause of congestive heart failure. Laboratory tests revealed abnormally low levels of total serum calcium (3.7 mg/dL), magnesium (1.7 mg/dL), and ionized calcium (0.6 mmol/L), with elevated phosphorus and creatinine levels and her brain natriuretic peptide (BNP) level was significantly elevated at 1855.1 pg/mL. Hormone analysis showed normal free T4 and thyroid‐stimulating hormone levels but decreased intact parathyroid hormone (PTH‐intact) and vitamin D levels. There was no sign of stenotic lesions on coronary angiography and therefore the patient was diagnosed with DCM due to severe hypocalcemia and idiopathic hypoparathyroidism. She took intravenous calcium and oral vitamin D_3_ and calcitriol during hospitalization. After discharge, she undertreated with oral calcium, vitamin D_3_, heart failure medications. Following treatment with intravenous calcium and oral vitamin D3 and calcitriol and normalizing the calcium levels, the patient's dyspnea significantly improved, and chest X‐ray showed reduced cardiomegaly and clearance of pulmonary edema, and the prolonged QT interval was shortened. Upon discharge, the patient continued with oral calcium, vitamin D3, and congestive heart failure medications. Follow‐up visits indicated gradual improvement in myocardial function, with the LVEF increasing to 46% within the first month and 51% by the third month.^9^
3. A 37‐year‐old woman with a history of thyroidectomy carried out at the age of 18, presented with cardiac symptoms. The surgery resulted in her developing permanent hypoparathyroidism. She was prescribed calcium and vitamin D supplements in addition to heart failure treatments, which she irregularly adhered to without undergoing regular laboratory monitoring. These symptoms included cramps, facial spasms, and heart irregularities. On examination cardiomegaly and pulmonary congestion were observed, indicating strain on the heart. An echocardiogram revealed dilatation and dysfunction of the left ventricle, mitral and tricuspid insufficiency, and moderate pulmonary hypertension. She also suffered from dyspnea, epigastric pain, and rapid edema progression. The patient's calcium levels were measured at 2.9 mg/dL, parathyroid hormone at 5.9 ng/L (normal range: 10–73), magnesium at 1.08 mEq/L (normal range: 1.4–2.1), hematocrit at 26%, hemoglobin at 7.4 g/dL, total creatine kinase at 1260 U/L (normal range: <70) (with a 3% myocardial fraction), and TSH at 18.1 µU/mL. The diagnosis indicated congestive heart failure secondary to severe hypocalcemia, decompensated by thyroid hormone replacement and exacerbated by iron‐deficiency anemia. The administration of calcium supplements, alongside diuretics, captopril, and digoxin, resulted in a swift and noticeable clinical enhancement. Subsequent monitoring for 18 months revealed the continued presence of LV enlargement and systolic dysfunction, although improvements were observed in all other echocardiographic observations and her cardiac symptoms improved significantly.^10^
4. A 70‐year‐old female presented to the emergency department with tonic‐clonic seizure. Her symptoms were paresthesia in her extremities for 2 months, altered sensorium for 1 month, exertional breathlessness, cough for 1 week, and three episodes of seizure 2 days before hospitalization. On the examination, Chvostek's sign, and Trousseau's sign were positive. The cardiovascular examination showed the presence of LV S3, while the respiratory examination revealed bilateral basal crepitations with rhonchi. Lab tests indicated significantly low serum calcium levels (3.4 mg/dL) and high serum phosphate levels (8.8 mg/dL), consistent with hypoparathyroidism. On her brain computed tomography scan, bilateral symmetrical calcification in the basal ganglia and dentate nucleus due to chronic hypoparathyroidism was revealed. The echocardiogram revealed severe DCM with congestive cardiac failure, characterized by LV systolic and diastolic dysfunction and an EF of 25%. Additionally, the patient's QT interval was prolonged (0.55 s), and she had mild mitral regurgitation, mild aortic regurgitation, and trivial tricuspid regurgitation. The final diagnosis was idiopathic hypoparathyroidism, severe hypocalcemia, and DCM with congestive cardiac failure. Treatment involved intravenous calcium gluconate and oral vitamin D_3_, and antiepileptic leading to remarkable improvement in the patient's condition in 2 weeks after normalization of calcium level.^11^
5. A 68‐year‐old woman presented to the emergency department due to intermittent dyspnea. She had recently undergone a subtotal thyroidectomy and was prescribed calcium supplementation. During the physical examination, she exhibited tachycardia and tachypnea. Chest radiography revealed bilateral pleural effusion, which was determined to be a transudate following aspiration. Blood tests indicated certain abnormalities: she had hypocalcemia (low calcium levels) at 1.15 mmol/L, high serum phosphate levels at 2.89 mmol/L, and a low PTH level, measuring less than 0.5 pmol/L. Additionally, transthoracic echocardiography (TTE) displayed dilation of the left ventricle and severe dysfunction, with an EF of only 15%, alongside moderate mitral and tricuspid regurgitation. The treatment included a β‐blocker, ACE inhibitor, spironolactone, calcium, and vitamin D. Within 2 weeks, electrolyte levels normalized, and the pleural effusion resolved. The EF improved to 35% after 3 weeks and 50% after 1 year.^5^
6. A 59‐year‐old woman presented at the cardiology outpatient clinic due to progressive exertional dyspnea and ankle edema. During the physical examination, a systolic murmur was detected, and NT‐pro‐BNP level unexpectedly was elevated. On her second visit to outpatient clinic, she had seizure and her brain CT scan revealed extensive calcifications in the basal ganglia. TTE revealed ventricular dilation with an estimated EF of 39%, along with moderate mitral regurgitation and severe tricuspid regurgitation. The ECG showed sinus rhythm with a leftward axis, a prolonged QTc interval of 533 ms, and ST depression in V4–V6. Laboratory tests indicated low plasma calcium levels (1.24 mmol/L) and elevated phosphate levels (2.5 mmol/L). The PTH level was undetectable (<0.6 pmol/L). Upon further investigation into the patient's history, it was discovered that she had been experiencing progressive muscle spasms and constipation for a few weeks. Ultimately, she received a diagnosis of hypocalcemia resulting from primary hypoparathyroidism. She suffered from DCM with congestive heart failure due to primary hypoparathyroidism. The patient received treatment consisting of vitamin D and intravenous and oral calcium supplementation. Within a week after achieving normal calcium levels, TTE revealed a notable improvement in the LV function.^5^
7. A 40‐year‐old woman with no cardiovascular history presented at the emergency care unit with symptoms including syncope, progressive exertional dyspnea, and paroxysmal nocturnal dyspnea. She had previously undergone thyroidectomy for Graves' disease and had type 1 diabetes mellitus. Physical examination revealed murmurs, gallop rhythm, peripheral edema, and lung stasis. The chest X‐ray demonstrated pulmonary stasis with hilum enlargement. The patient's ECG showed sinus tachycardia with a prolonged QT interval along with negative T waves from V1 to V4. Echocardiography revealed dilated left chambers and severe global systolic dysfunction of the left ventricle (LVEF = 15%) due to diffuse hypokinesia. Restrictive diastolic dysfunction with elevated filling pressure indexes, along with moderate functional mitral and tricuspid regurgitation and moderate pulmonary hypertension, were also observed. Angio coronarography ruled out atherosclerotic lesions. Laboratory blood tests indicated severe hypocalcemia, with a total serum calcium level of 3.6 mg/dL. Other lab findings were demonstrated high blood sugar, elevated TSH and NT‐pro‐BNP levels. Further assessment involving parathormone testing, phosphoric, and other related examinations confirmed a diagnosis of severe primary hypoparathyroidism. At first, she treated with ACE inhibitor, beta‐blocker, anti‐aldosterone diuretic, insulin, atorvastatin, and levothyroxine. During hospitalization, she exhibited hypocalcemia signs such as Chvostek, Weiss, Trousseau positive. The brain CT‐scan revealed calcifications of the basal ganglia. She took gluconic calcium at first and then was treated with oral lactic calcium and calcitriol. The patient received calcium treatment, both parenteral and oral, which led to improved clinical and serological outcomes. After 1 month, the patient was discharged with significant improvement in cardiac symptoms and LVEF. One year later, the patient remained on medication, experiencing only dyspnea during intense efforts, with normal calcium and vitamin D levels. Follow‐up cardiac tests showed normal results.^12^
8. A 29‐year‐old woman was admitted to our hospital presenting with congestive heart failure characterized by severe dyspnea (NYHA class IV) and generalized edema that had persisted for 2 days. She had a history of undergoing a total thyroidectomy 1 year prior and was prescribed daily Levothyroxine medication along with calcium supplementation and vitamin D due to her hypoparathyroidism although she did not consistently adhere to her treatment. During the physical examination, her blood pressure was measured at 90/60 mmHg. The chest examination revealed crackling sounds in both lung areas, a 3/6 systolic murmur at the apex of her heart, and a galloping rhythm (S3). A chest X‐ray displayed an enlarged heart with fluid accumulation in the lungs. The ECG showed signs of sinus tachycardia, an extended QT interval, and negative T‐waves in specific leads. Laboratory tests indicated that her corrected calcium levels were as low as 3.2 mg/dL (normal range: 8.5–10.5 mg/dL), and her ionized calcium was 0.36 mmol/L (normal range: 0.85–1.05). Her BNP levels were elevated at 580 pg/mL (normal range: 100 pg/mL) and her intact parathyroid hormone (PTH‐I) levels were low at 10.50 pg/mL (normal range: 16–68 pg/mL). TTE revealed a dilated left ventricle with global hypokinesia and a reduced LVEF of 28%, in addition to moderate mitral regurgitation. Treatment began with dobutamine, furosemide, calcium, and vitamin D3, and an angiotensin‐converting enzyme inhibitor (ACEI) and beta‐blockers. Following the correction of the hypocalcemia, a significant improvement in the patient's clinical condition was observed, and after 5 months of treatment, the patient's condition significantly improved. A follow‐up chest X‐ray displayed normal results (no enlarged heart or signs of lung congestion) and Echocardiography revealed a normal‐sized LV with a healthy ejection function (ranging from 28% to 63%).^13^
9. A 44‐year‐old man was referred to the hospital due to resistant congestive heart failure accompanied by dyspnea and lower limb edema, despite receiving optimal treatment. The patient had no previous medical history. Upon admission, the physical examination revealed a blood pressure of 90/50 mmHg, tachycardia (heart rate of 118 bpm), tachypnea with orthopnea, crackling lung sounds extending to the upper lung regions, and bilateral lower limb swelling. The ECG showed a sinus rhythm with inverted T waves in the precordial leads. Chest X‐ray findings indicated an enlarged heart with increased vascularity in two lung fields. Laboratory tests revealed corrected calcium levels of 4.5 mg/dL (normal range: 8.5–10.5 mg/dL), ionized calcium at 0.43 mmol/L (normal range: 0.85–1.05) and Hormone assays showed low PTH‐I levels at 8.50 pg/mL (normal range: 16–68 pg/mL). TEE revealed DCM with a significant impairment in LV systolic function (25%). The diagnosis established was DCM caused by hypocalcemia due to primary hypoparathyroidism. Treatment involved oral calcium, vitamin D, ACE inhibitors, and beta‐blockers (bisoprolol). After 5 months, a full recovery of the cardiomyopathy was observed, and the patient became symptom‐free (with no edema or dyspnea). Follow‐up chest X‐rays showed normal results, and ETT revealed a normal‐sized left ventricle with a normal EF (ranging from 25% to 60%).^13^
10. A 60‐year‐old male was admitted to the hospital with severe pulmonary congestion, recurrent pulmonary edema, and pleural effusion. He had a history of primary DCM and received an implantable cardioverter‐defibrillator. However, his condition did not respond to standard heart failure treatment. Laboratory tests revealed low blood calcium levels, high phosphate levels, and decreased PTH activity. Elevated levels of creatine phosphokinase (CPK) and BNP were observed. ECG showed a prolonged corrected QT interval (QTc). Echocardiography confirmed left ventricle dilatation, reduced EF of 20%, and extremely decreased global longitudinal strain (GLS). The patient had a previous subtotal thyroidectomy 36 years ago. Treatment with calcium and vitamin D supplementation led to a significant improvement in symptoms and cardiac function. The patient was discharged after 4 weeks and showed continued improvement during the follow‐up.^14^
11. A 26‐year‐old woman was diagnosed with hypoparathyroidism, characterized by symptoms of paresthesia and upper limb convulsions. Despite the diagnosis, she did not adhere to prescribed medication or medical check‐ups due to limited intelligence. Approximately 1 year after the diagnosis, she was admitted to the hospital with acute shortness of breath. Physical examination revealed a short stature, malnourished appearance, and severe tooth decay. Blood pressure was low, and the heart rate was regular. ECG showed a prolonged QT interval. Echocardiography revealed a dilated left ventricle with severely reduced EF (25%) due to diffuse hypokinesia and impaired diastolic function. Cardiac magnetic resonance did not reveal any signs of myocardial inflammation or replacement fibrosis. Laboratory tests indicated high NT‐pro‐BNP level, advanced hypocalcemia, hypomagnesemia, and hyperphosphatemia, along with signs of renal and liver dysfunction. The diagnosis of acute heart failure due to hypo‐calcemia associated with untreated hypoparathyroidism was made. Treatment included heart failure therapy, calcitriol administration, and calcium and magnesium supplementation. Upon discharge, the patient was asymptomatic, and blood ion levels had normalized. Nevertheless, a year after being discharged, there was a recurrence of low calcium and magnesium levels, and the patient's heart function worsened because she failed to adhere to treatment. The outlook for her long‐term health remains unclear.^15^
12. A 74‐year‐old man with a history of arterial hypertension, stage 3 chronic kidney disease, prostate cancer treated with curative radiation in 2013, and a complete thyroidectomy for goiter in 2009 are among his past medical conditions. He was not given any treatment despite having low plasma calcium levels in 2014. He was hospitalized in June 2017 due to dyspnea. Upon workup, DCM with an EF of 44%, hyperphosphatemia, and hypocalcemia were found. Due to primary hypoparathyroidism, he was diagnosed with hypo‐calcemic DCM. The patient was started on heart failure medicine and calcium supplements; however, the patient did not comply with the treatment plan. He was admitted to the hospital several times in the following months due to decompensation of heart failure. His EF dropped to 26% in June 2018, and he experienced acute renal damage that necessitated hemodialysis. Lab tests revealed new hypomagnesaemia, hyperphosphatemia, and increasing hypocalcemia. The hypoparathyroidism treatment was resumed, and results improved. In September 2018, during the 3‐month follow‐up, he reported taking his medicine as prescribed. His EF increased to 52%, and his calcium and phosphorus levels had returned to normal.^15^

### 3.1 Diagnosis

DCM, hypertrophic cardiomyopathy, arrhythmia, and congestive heart failure are among the most common cardiac involvements that hypoparathyroidism can cause.^17^, ^18^ The precise cardiac involvement in hypoparathyroidism is influenced by hypocalcemia, which can result in decreased myocardial tension, expansion of the cardiac cavity, and arrhythmia.^11^, ^18^

Acute, temporary, DCM with low EF and widespread LV hypokinesia is known as hypo‐calcemic cardiomyopathy. It is linked to calcifications in the brain.^18^ Prolonged hypoparathyroidism can result in LV dysfunction and arrhythmia.^19^ LV systolic dysfunction caused by hypoparathyroidism can result in a reversible DCM.^11^ Moreover, a rare condition known as chronic hypoparathyroidism‐associated cardiomyopathy may result in decreased myocardial tension, expansion of the ventricular cavity, arrhythmia, and congestive heart failure.^18^

The clinical and laboratory findings are used to make the diagnosis of hypoparathyroidism. Muscle cramps, paresthesia, seizures, and neuropsychiatric complications are among the usual symptoms of hypoparathyroidism, though the clinical presentation may differ widely. Low serum calcium levels, high serum phosphate levels, and low or improperly normal PTH levels are common laboratory results.^11^

The diagnosis of DCM is made using clinical and imaging data. Typically, echocardiography is used to make the diagnosis. This test may reveal LV enlargement and a decreased EF.^20^ DCM can also be identified using other imaging techniques, such as cardiac computed tomography (CT) scans and cardiac magnetic resonance imaging (MRI).^21^ In some cases, to confirm the diagnosis, endo‐myocardial biopsy is essential.^22^

Therefore, clinical and laboratory findings are used to determine whether hypoparathyroidism is the cause of cardiac involvement. Patients with hypoparathyroidism who experience cardiac involvement can suffer from symptoms including syncope, palpitations, dyspnea, and chest pain.^15^, ^17^ Low serum calcium levels, high serum phosphate levels, and elevated cardiac biomarkers like troponin and BNP are potential laboratory findings.^15^ Although ECG findings in DCM cannot be specific changes and can be related to other cardiac conditions, DCM can present with varying degrees of ST‐T wave changes, QRS voltage, prolonged QT interval, and sinus tachycardia on the ECG, reflecting myocardial dysfunction and potential myocarditis.^17^ Conduction abnormalities, such as bundle branch blocks or other types of intraventricular conduction delays, can also be related to DCM.^7^ Also, echocardiography can reveal hypo‐calcemic cardiomyopathy, a transitory, acute, DCM associated with lower EF and LV hypokinesia, in hypoparathyroidism.^7^, ^15^, ^17^

## 4 MANAGEMENT

Given that hypocalcemia is the primary cause of cardiac symptoms in hypoparathyroidism, treatment focuses on correcting serum calcium levels. Ensuring proper serum calcium balance is considered the central aspect of managing the condition and addressing associated cardiac issues. The treatment of hypocalcemia in hypoparathyroidism is not solely determined by calcium levels but also takes into account associated symptoms. Patients with hypoparathyroidism may exhibit chronically low‐normal or mildly hypo‐calcemic levels. The severity of symptoms and signs, such as paresthesia, carpopedal spasm, broncho‐ or laryngospasm, tetany, seizures, mental status changes, Chvostek's or Trousseau's signs, bradycardia, impaired cardiac contractility, and prolongation of the QT interval, varies based on the absolute level of calcium.^23^ Chronic hypocalcemia is managed with oral calcium and vitamin D supplements; acute forms may require intravenous calcium.^24^ Immediate treatment for symptomatic hypocalcemia involves administering intravenous Ca^2+^ salts in two stages. Initially, one or two ampules of a 10% calcium gluconate solution, containing 90–180 mg elemental calcium in 50 mL of 5% dextrose, are given over 10–20 min. This is followed by a slower infusion of calcium gluconate, ranging from 0.5 to 1.5 mg/kg/h, administered over 8–10 h.^23^ For the management of chronic hypocalcemia, calcium carbonate, and calcium citrate, with high elemental calcium content, are preferred supplements. Dosages of 1–2 g of elemental calcium, three times daily, are recommended and can be adjusted as needed.^25^ In addition to calcium supplementation, the treatment of hypoparathyroidism necessitates the use of vitamin D analogs orally. Active vitamin D metabolites like Calcitriol (1,25 (OH) 2D3) or alfacalcidol (1α (OH)D 3) are recommended due to their potency and longer duration of action, while colecalciferol is not recommended. For adults, a dose of 1–1.5 mcg alfacalcidol along with calcium carbonate should be used to maintain normal serum calcium and phosphate levels.^24^, ^26^

It is advisable to avoid loop diuretics in cases of heart failure due to hypoparathyroidism, as they may exacerbate the loss of calcium in urine.^27^ Thiazide diuretics, especially when used in conjunction with a low‐salt diet, can effectively reduce urinary calcium excretion.^28^ Therefore, it is recommended to consider prescribing hydrochlorothiazide (25 mg per day) and spironolactone (20 mg per day) to help alleviate heart failure symptoms. Alongside this, following heart failure guidelines, the use of Sacubitril Valsartan Sodium Tablets and Metoprolol Succinate Sustained‐release Tablets is recommended, with their dosages adjusted for optimal tolerance.^27^

**Figure 1:**
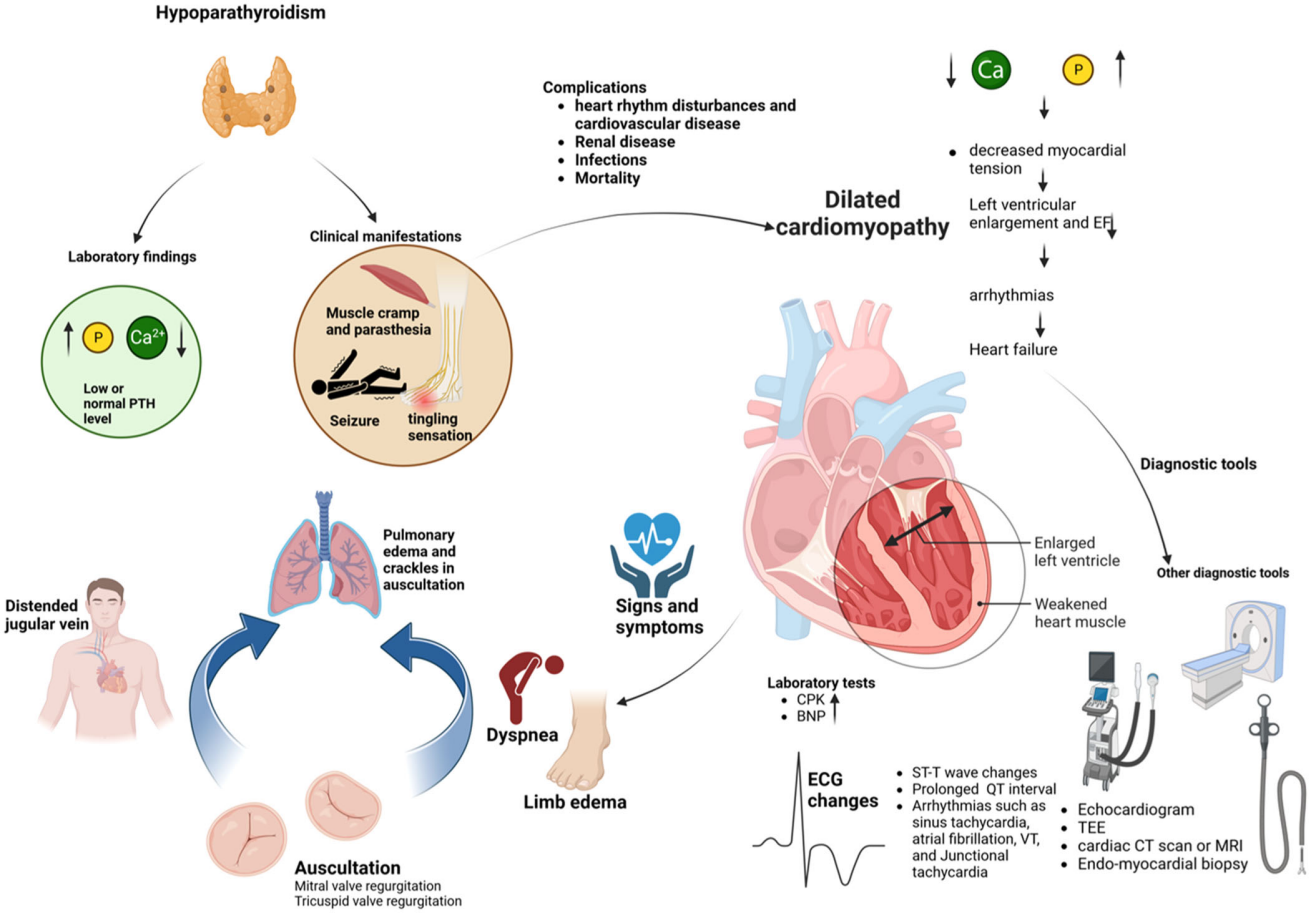
This figure demonstrates cardiac involvement, diagnosis, and management in patients with hypoparathyroidism.

Furthermore, it's crucial to emphasize that, as previously mentioned, noncompliance with treatment is a significant factor contributing to cardiac complications in hypoparathyroidism. Non‐adherence is a significant issue that has far‐reaching impacts, affecting both the patient and the healthcare system. When patients fail to adhere to their medications, it results in the worsening of diseases, increased mortality, and higher healthcare expenses.^29^ Adherence rates for chronic conditions typically range from 50% to 60%, even among insured patients who have access to affordable generic medications. Noncompliance with medical recommendations can be attributed to various factors, including patient‐centered aspects like demographics, psychosocial factors, and health literacy. Treatment‐related factors, such as complexity and side effects, also play a role, along with healthcare system challenges. Additionally, social and economic factors, as well as the nature of disease symptoms, influence patient adherence.^30^, ^31^ Studies show that physicians can significantly impact health outcomes by discussing medication adherence and involving patients in decision‐making. Simplified regimens and improved communication are perceived as effective adherence strategies.^32^ Healthcare reforms, such as addressing financial barriers, embracing technology, and customizing adherence programs for high‐risk patients, may be necessary.^33^ Cardiac involvement, diagnosis, and management in patients with hypoparathyroidism are shown in Figure 1.

## 5 DISCUSSION

Hypocalcemia is a rare but recognized contributor to DCM (CMP). The precise mechanisms behind hypo‐calcemic CMP are not yet fully understood, despite our knowledge of calcium's crucial role in muscle contraction. When the cell membrane depolarizes, calcium ions enter muscle cells via L‐type calcium channels, triggering muscle contraction by releasing calcium from the sarcoplasmic reticulum and facilitating the interaction of actin and myosin filaments. Experimental evidence has shown that hypocalcemia can lead to diminished activation of the calcium‐sensing receptor, decreased heart muscle contractility, and an extension of the QT interval.^34^, ^35^

In these cases of DCM related to hypoparathyroidism, a consistent pattern emerges, with patients often presenting with symptoms of heart failure, such as dyspnea, palpitations, and edema. The cardiac manifestations are characterized by reduced EF, ventricular dilation, and impaired LV function. Alongside these cardiac abnormalities, laboratory tests reveal low serum calcium levels and high phosphate levels, confirming the presence of hypocalcemia. Moreover, ECGs often show prolonged QT intervals. The patient's medical history commonly includes thyroid surgery, such as total or subtotal thyroidectomy.

A notable observation in these cases is the poor compliance of patients in taking their prescribed medications and calcium supplements. Nonadherence to treatment has been identified as a significant factor contributing to the development and exacerbation of cardiac complications in these individuals. Healthcare providers must address this issue during patient consultations and stress the importance of consistent medication intake to maintain stable calcium levels and prevent cardiac‐related complications.

Indeed, an important feature in these cases is the absence of significant coronary artery disease, as confirmed by angiography. The cardiac dysfunction observed in DCM related to hypoparathyroidism appears to be primarily linked to the disruption of calcium homeostasis and not due to traditional atherosclerotic processes seen in coronary artery disease. This further emphasizes the critical role of hypocalcemia in the pathogenesis of DCM in these patients.

Successful management of the condition involves restoring calcium and vitamin D levels, which results in significant improvements in cardiac symptoms and function.^36^ These cases underscore the critical role of calcium in maintaining normal cardiac function. Hypocalcemia due to hypoparathyroidism disrupts the delicate balance of calcium ions necessary for proper cardiac muscle contraction and electrical signaling, leading to the development of DCM.^37^ Prompt identification and appropriate treatment of hypocalcemia are crucial in reversing cardiac dysfunction and improving the patient's overall prognosis.

Response to treatment can be effectively monitored through a combination of blood check‐ups, electrocardiography (ECG), and echocardiography. These diagnostic tools allow healthcare providers to track the response to treatment and make necessary adjustments. Healthcare providers must emphasize the importance of medication adherence to ensure the best possible outcomes for patients.

## 6 CONCLUSION

The cases presented showcase the potential for hypoparathyroidism to result in DCM, a reversible form of heart failure. Several notable commonalities emerge across these cases. Most patients exhibited symptoms of heart failure like dyspnea, edema, and fatigue. Echocardiographic findings revealed impaired LV function and reduced EF. Hypocalcemia was consistently present, evident through laboratory testing, alongside other electrolyte imbalances like hyperphosphatemia.^15^, ^17^

The underlying mechanism relates to the critical role of calcium in myocardial contraction. Calcium influx is essential for actin‐myosin crossbridge formation and subsequent cardiac muscle contraction.^16^ Hypocalcemia impairs this process, reducing myocardial contractility and cardiac output.^15^ Structural changes like ventricular dilation and hypokinesia occur over time. Electrophysiological abnormalities also arise, disrupting normal electrical conduction and prolonging QT interval.^18^

For clinicians, these cases demonstrate the importance of considering hypocalcemia and hypoparathyroidism as potential reversible causes of heart failure.^11^ Monitoring calcium levels and prescribing calcium and vitamin D supplementation are vital for management.^23^, ^24^ Patient education on medication adherence is also key to prevent complications.^12^, ^13^ With prompt diagnosis and treatment of the electrolyte imbalance, cardiac function can improve significantly, underscoring the potential for reversibility in this form of DCM.^7^ Routine screening for hypocalcemia in those with risk factors like neck surgery can allow for earlier detection and treatment.^4^

In summary, the presented cases provide valuable insights into hypocalcemic DCM. They highlight the underlying pathophysiology relating calcium to contractility, while also emphasizing key clinical considerations in diagnosis and management. A dedicated discussion section can effectively synthesize the case information to provide a cohesive overview.

The link between hypoparathyroidism and DCM is evident in the presented cases, where patients exhibited cardiac symptoms and abnormal echocardiographic findings. The absence of significant coronary artery disease in these patients further emphasizes the central role of hypocalcemia in DCM development.

Notably, these cases highlight the importance of patient compliance with prescribed medications and calcium supplements to prevent and manage cardiac complications. patient education plays a vital role in managing hypoparathyroidism and its associated cardiac complications. It is essential to inform patients about the potential dangers of noncompliance with prescribed medications and calcium supplements. Regular follow‐up check‐ups are equally important to monitor calcium levels and overall health status.

By emphasizing the significance of adhering to treatment regimens, we can help patients understand the potential risks of untreated hypocalcemia and its impact on cardiac function. Encouraging consistent intake of calcium and vitamin D supplements, along with prescribed medications, can significantly reduce the risk of developing DCM and other cardiac symptoms.

Regular check‐ups and monitoring of calcium levels can provide valuable insights into the patient's condition, helping healthcare providers detect any fluctuations in calcium levels and promptly address potential complications. Additionally, as DCM is seen in some patients with any type of endocrine disorders including hypothyroidism, hypoparathyroidism, cushing disease, and so on, we recommend routine follow‐up of DCM among these patients.

### 6.1 Limitations

This study has potential limitations. The effect discussed in the manuscript are based on available case reports and there is a huge lack of research in patients with hypoparathyroidism who develop DCM. They are therefore subject to biases and confounding that may have influenced our paper.

We encourage all physicians to have a regularly follow‐up of cardiac examinations in patients with hypoparathyroidism. Therefore, we can have more information about DCM in patients with hypoparathyroidism to conclude the exact relationship, reduce the rate of morbidity, and prevent cardiac involvement before DCM will appear.

## AUTHOR CONTRIBUTIONS

**Maryam Abdolmaleki**: Conceptualization; writing—original draft; writing—review and editing. **Laya Ohadi**: Conceptualization; writing—original draft; writing—review and editing. **Saba Maleki**: Visualization; writing—original draft.

## CONFLICT OF INTEREST STATEMENT

The authors declare no conflict of interest.

## TRANSPARENCY STATEMENT

The lead author Laya Ohadi affirms that this manuscript is an honest, accurate, and transparent account of the study being reported; that no important aspects of the study have been omitted; and that any discrepancies from the study as planned (and, if relevant, registered) have been explained.

## Data Availability

Data are available on request from the authors.

## ENDNOTES

^1^
New York Heart Association Functional Classification/IV: Severe limitations. Experiences symptoms even while at rest.^2^ Shortness of breath when lying down.
